# Host modulation therapy in periodontitis: from established therapies to emerging technologies

**DOI:** 10.3389/fimmu.2026.1762187

**Published:** 2026-02-20

**Authors:** Aonjittra Phanrungsuwan, Jiaqi Huang, Neeraja Dharmaraj, Alejandra Cobos Perez, Omid Veiseh, Simon Young, Chun-Teh Lee

**Affiliations:** 1 Department of Periodontics and Dental Hygiene, School of Dentistry, The University of Texas Health Science Center at Houston, Houston, TX, United States; 2 Katz Department of Oral and Maxillofacial Surgery, School of Dentistry, The University of Texas Health Science Center at Houston, Houston, TX, United States; 3 Department of Bioengineering, Rice University, Houston, TX, United States

**Keywords:** cytokines, drug delivery systems, genetics, microbial, immunomodulation, periodontitis

## Abstract

Periodontitis is a biofilm-induced chronic inflammatory disease, characterized by gingival inflammation and alveolar bone loss. According to a national survey, approximately half of the U.S. adults are affected by periodontal disease. To effectively prevent and treat periodontitis, it is essential to address its underlying causes. The primary etiological factors include polymicrobial synergy and dysbiosis of the oral microbiota, and a dysregulated immune response. The standard therapeutic approach, mechanical removal of biofilm through debridement, sometimes demonstrates limited efficacy, particularly in cases of severe periodontitis, which may require adjunctive or additional therapy. Emerging evidence indicates that periodontal tissue destruction is initiated by biofilm but primarily driven by a sustained, dysregulated host inflammatory response characterized by excessive cytokine production, osteoclast activation, and impaired inflammation resolution. In recent years, research has focused on targeting both the oral microbiota and immune response by utilizing antimicrobial therapeutics to diminish bacterial load and by modulating immune activity. Specifically, host modulation therapies (HMTs), such as the delivery of anti-inflammatory cytokines, nonsteroidal anti-inflammatory drugs (NSAIDs), low-dose doxycycline, and lipid mediators, via various techniques, have been explored. However, challenges associated with its use encompass adverse effects resulting from prolonged administration, and systemic delivery methods are associated with an elevated risk of infection and the potential development of malignancy, as well as the disease rebound after cessation of treatment. This review examines current trends in HMTs for periodontitis and identifies potential limitations of these approaches. The insights gained may contribute to the development of improved strategies to enhance periodontal treatment outcomes.

## 1 Introduction

Periodontitis is a chronic inflammatory disease induced by biofilm accumulation, characterized by gingival inflammation and alveolar bone loss surrounding the teeth. It is the primary cause for tooth loss, affecting nearly half of the population aged 30 years and older (approximately 60.7 million individuals) in the United States. Specifically, severe periodontitis affects over one billion people globally (1–3). The global economic burden attributable to productivity losses resulting from periodontitis has been estimated at approximately $54 billion per year in direct treatment costs (2–4). It is critical to address the underlying pathological mechanisms to prevent and treat periodontitis. The main causes of periodontal diseases stem from bacterial dysbiosis and are further mediated by the host response to the bacteria (5–7). Although initiated by microbial dysbiosis, the progression and severity of periodontitis are largely determined by an exaggerated and unresolved host immune response, marked by excessive pro-inflammatory cytokine production, osteoclastogenesis, and failure of inflammation resolution pathways. For decades, mechanical debridement has been the primary method for biofilm removal in periodontal therapy. While effective in many patients, this strategy often demonstrates limited success in individuals with advanced or aggressive disease phenotypes, where hyperinflammatory host responses persist despite microbial control. Such observations underscore the critical role of immune dysregulation in periodontal disease progression and highlight the limitations of therapies that target bacteria alone. Therefore, restoring an appropriate host immune response is crucial for effective treatment; however, there is currently no established host modulation therapy (HMT) has proven to be effective. Therefore, over the past two decades, there has been a growing trend towards developing new therapies that target the exacerbation of the immune response in patients with periodontitis. Non-steroidal anti-inflammatory drugs (NSAIDs) have been utilized in the management of periodontitis, demonstrating some favorable clinical outcomes (8–10). However, there are significant concerns about long-term use because of multiple adverse effects, and the rebound of disease after stopping treatment has become a major drawback (11). Systemic anti-cytokine therapies, targeting specific cytokines to reduce inflammation, have been extensively investigated; however, their efficacy appears to be confined to periodontitis patients with underlying autoimmune disorders. Moreover, concerns exist regarding potential adverse effects, including an increased risk of infection and malignancy (12, 13).

In recent years, we have witnessed the rapid development of utilizing engineered viruses and bacteria, such as *Limosilactobacillus reuteri* (*L. reuteri;* previously known as *Lactobacillus reuteri*), *Lactobacillus plantarum*, and *Lactobacillus brevis*, to treat inflammatory diseases. These organisms are lactic acid bacteria (LAB) colonizing the mammalian gastrointestinal tract and oral cavity and serve as a delivery vector for anti-cytokine agents (14). For example, *L. reuteri* exhibits a range of probiotic properties, notably by attenuating the synthesis of pro-inflammatory cytokines and facilitating the development and functioning of regulatory T cells (15). It is utilized in the treatment of cancer immunotherapy as well as chronic inflammatory diseases, including inflammatory bowel disease, Crohn’s disease, and tissue fibrosis, in both pre-clinical and clinical studies (16–20). Furthermore, LAB administration has been demonstrated to mitigate bone loss in murine periodontitis models (21). In clinical investigations, oral intake of *L. reuteri* has been associated with decreased levels of periodontal pathogens and an inhibitory effect on periodontal inflammation (22–24), without eliciting adverse effects. However, these organisms are not yet widely used in clinical practice for treating oral diseases. A significant obstacle is the inability to effectively deliver and retain these bacteria within diseased tissues *in vivo*. This review focuses on the current use of HMT in inflammatory diseases, especially periodontitis, and explores the challenges linked to HMT. It also covers innovative technologies such as engineered bacteria, which could help bridge gaps in translation, improve therapeutic approaches, and deepen our understanding of this emerging field.

## 2 HMT in inflammatory diseases and cancer

An imbalance in host immune function causes several diseases, including Crohn’s disease, tissue fibrosis, rheumatoid arthritis, diabetes mellitus, and various types of cancer. HMT constitutes a therapeutic paradigm that endeavors to modify the physiological state or functional capacity of the host organism as a strategic approach to disease management. Over the last decade, extensive experiments have been conducted on various immunomodulatory and microbiome-based strategies to combat inflammation-driven and immune-related diseases. Recently, novel approaches have demonstrated promising results. Zanin-Zhorov and Blazar reviewed the pivotal role of Rho-associated coiled-coil containing protein kinase 2 (ROCK2) in immune dysregulation and fibrosis in chronic graft-versus-host disease (cGVHD), highlighting how its selective inhibition via belumosudil not only restored Treg/Th17 balance but also showed over 70% response in refractory cGVHD patients (25). In parallel, Lu et al. emphasized Serum and Glucocorticoid Regulated Kinase 1 (SGK1) as another fibrosis-driving molecule in cGVHD, influencing T-cell differentiation and promoting tissue fibrosis. The use of SGK1 inhibitors as an immune modulator has shown promise in reversing fibrotic changes (26). As for cancer immunotherapy, Gao et al. found that the probiotic *Lacticaseibacillus rhamnosus* Probio-M9 enhanced anti-Programmed Cell Death Protein-1(PD-1) efficacy in colorectal cancer by modulating gut microbiota, enriching beneficial microbes, and increasing tumor-infiltrating cytotoxic T-cells (27). Complementing this, Fong et al. reviewed broader strategies, such as probiotics, prebiotics, and fecal microbiota transplantation (FMT), for colorectal cancer prevention and treatment, emphasizing the dual roles of microbiota in modulating inflammation and chemoresistance (28). Focusing on cystic fibrosis, Bedi et al. uncovered how *Pseudomonas aeruginosa* exploits endoplasmic reticulum stress pathways, particularly via CCAAT/enhancer-binding protein homologous protein (CHOP)-mediated suppression of peroxisome proliferator-activated receptor gamma (PPARγ), to impair epithelial host defenses, and proposed PPARγ agonists, such as pioglitazone, to restore immune balance (29). Gowen et al. underscored the potential of precision microbiome therapeutics, discussing how interkingdom microbial interactions and biofilm dynamics influence host immunity and suggesting personalized probiotic development as a next-generation disease treatment strategy (30).

Despite promising outcomes, significant challenges remain in the application of immune-targeted treatments. Pathway inhibitors, such as ROCK2 and SGK1, show therapeutic potential in cGVHD, yet their broad roles in tissue homeostasis and metabolism raise concerns about off-target and systemic effects, necessitating isoform-specific targeting and dose refinement (25, 26). In cancer immunotherapy, microbiota modulation via probiotics or fecal transplantation enhances efficacy but faces hurdles of strain-specificity, variable host microbiomes, pathogen transmission, and regulatory oversight, underscoring the need for personalized and standardized approaches (27). Novel strategies, such as β–glucan–induced trained innate immunity, highlight the difficulty of balancing enhanced host defense with the risk of autoimmunity. Meanwhile, metabolic regulators, including PPARγ agonists in cystic fibrosis, carry systemic side effects that limit their chronic use. Furthermore, the complexity of microbiota–host interactions, including biofilm dynamics and interkingdom signaling, complicates the predictability of responses in diseases like Crohn’s, emphasizing the necessity for precision-engineered and scalable microbial therapeutics (29–31).

While these approaches appear promising, it can be challenging to anchor these advances to oral diseases because of the distinct immunological and microbial landscape of the oral environment. The oral cavity is characterized by constant mechanical stress, a polymicrobial biofilm, and a rapidly responsive and complex immune environment. While all challenges, such as systemic effects from pathway modification, interindividual variability, and durability of probiotic colonization, remained, it is even more difficult to balance microbiota and immune responses to achieve immune modulation without disrupting oral mucosal homeostasis. Collectively, while systemic inflammatory disease models provide valuable mechanistic frameworks, successfully adapting these therapies for oral diseases will require careful consideration of the specific immunological and microbial conditions present in the oral cavity.

## 3 HMT in periodontitis

In the context of periodontitis, HMTs have been investigated for nearly three decades to counteract microbial dysbiosis and attenuate destructive inflammatory processes. This therapeutic approach focuses on modulating the host immune response to foster an environment conducive to a balanced oral microbiome while suppressing the exaggerated inflammatory reactions that contribute to periodontal tissue breakdown (32–35). Various strategies have been employed to modulate the immune response, aiming to reduce tissue damage, maintain a balanced environment between pro-inflammatory and anti-inflammatory mediators, and promote the resolution of inflammation and periodontal tissue repair (34).

### 3.1 The use of NSAIDs

NSAIDs inhibit cyclooxygenase (COX) enzymes, reducing prostaglandin E_2_ (PGE_2_) production, which is elevated in inflamed periodontal tissues and linked to vasodilation and bone resorption by inducing nuclear factor kappa-B ligand (RANKL) production from activated B and T cells as well as osteoblasts (36). Clinical trials and animal models of periodontitis have demonstrated that mechanical therapy, combined with NSAID treatment, enhances clinical outcomes, including reductions in gingival inflammation, clinical attachment loss, and bone loss. However, it is associated with disease rebound following drug discontinuation, and the long-term application is contraindicated due to potential gastrointestinal and cardiovascular risks (9, 18, 37–39).

### 3.2 Specialized pro-resolving mediators

While NSAIDs are associated with significant limitations when employed in long-term therapy, over the past two decades, there has been an increasing interest in utilizing SPMs in the treatment of periodontal diseases. SPMs, including lipoxins (e.g., LXA4), resolvins (e.g., RvE1, RvE2), maresins, and protectins, are bioactive lipid compounds derived from omega-3 and omega-6 fatty acids. *In vivo* studies utilizing rabbit and rat models of periodontitis have demonstrated that RvE1 can inhibit tissue and alveolar bone destruction by over 95%. Additionally, RvE1 administration has been shown to facilitate tissue regeneration, including restoring the periodontal ligament and reversing alveolar bone loss (40, 41). SPMs play a crucial role in the resolution of inflammation by modulating immune responses. For instance, they enhance pathogen clearance through activation of neutrophils and macrophage phagocytosis and mitigate tissue damage by preventing neutrophil overactivation through reducing beta-2 integrin expression and intercellular adhesion molecule 1, and upregulating the level of nitric oxide (42). Hasturk et al. conducted a clinical trial using a mouthwash containing the LXA4 analog, methyl ester-bezo-lipoxin A4 (BLXA4), for patients with gingival inflammation. Once-daily rinsing with BLXA4 for 28 days significantly reduced gingival inflammation and pocket depths compared to the placebo and no-rinse groups. Although preliminary pre-clinical and clinical results demonstrate promising potential for treating periodontitis, extensive longitudinal clinical studies are necessary before its implementation in routine clinical practice (43).

### 3.3 Sub-antimicrobial dose doxycycline

Low-dose doxycycline is currently the only FDA-approved host-modulating drug specifically indicated as an adjunct to periodontal therapy. Administered at sub-antimicrobial levels (commonly 20 mg twice daily), SDD has been shown to provide modest but consistent clinical benefits, including approximately 0.3–0.4 mm greater clinical attachment gain beyond scaling and root planning (SRP) alone, along with reduced inflammation (44–46). Its primary mechanism of action involves inhibition of matrix metalloproteinases (MMPs), particularly MMP-8 and MMP-13, thereby limiting collagen degradation and connective tissue breakdown. Importantly, clinical trials demonstrate that up to 12 months of SDD use do not significantly alter the subgingival microbiota or increase antibiotic resistance (44, 46). However, several limitations remain. The long-term outcomes beyond one year are insufficiently documented, and the drug’s narrow mechanism of action does not address the broader inflammatory networks driving periodontitis. Furthermore, issues related to patient compliance, cost, and systemic exposure may restrict its widespread application in routine clinical practice (47).

### 3.4 Cytokine therapy

Cytokines serve as critical mediators in the signaling processes that regulate the proliferation and activation of both innate and adaptive immune responses. The therapeutic application of cytokines has been extensively employed in various clinical contexts, including cancer immunotherapy, autoimmune disorders, infectious diseases, and other immunological conditions (48, 49). In periodontal diseases, therapeutic strategies include the use of neutralizing monoclonal antibodies or receptor antagonists to inhibit proinflammatory cytokines. For instance, the inhibition of interleukin-1 (IL-1), IL-17, and tumor necrosis factor-alpha (TNF-α) has been associated with a reduction in alveolar bone loss. These cytokines are expressed on both osteoclast precursor cells and mature osteoclasts. Both *in vitro* and *in vivo* studies have demonstrated that proinflammatory cytokines play a pivotal role in osteoclastogenesis by regulating the production of receptor activator of RANKL and osteoprotegerin (OPG) (50–52). Recent research has demonstrated an increasing interest in the role of anti-inflammatory cytokines (53, 54). IL-10, in particular, is recognized as a potent anti-inflammatory cytokine that suppresses both immunoproliferative and inflammatory responses. IL-10 exerts its effects by downregulating the synthesis of pro-inflammatory cytokines and chemokines, such as IL-1, IL-6, and TNF-α (55, 56). A deficiency in IL-10 levels has been associated with inadequate inhibition of pro-inflammatory cytokines and collagenases. *In vivo* studies have further corroborated that IL-10 upregulates OPG expression while concurrently downregulating the receptor activator of RANKL and colony-stimulating factor-1 (CSF-1) (57, 58). Beyond its inhibitory effects on osteoclastogenesis, IL-10 has also been shown to promote osteoblastic differentiation *in vivo*. The application of anti-inflammatory cytokines in models of periodontitis and the related outcomes represents significant areas for future research (53). Nevertheless, it is important to recognize that targeting individual cytokines may alter multiple downstream signaling pathways, potentially resulting in unintended consequences, including increased susceptibility to infections and other adverse effects. In addition, periodontal tissue destruction is regulated by complex and redundant cytokine networks, which may limit the effectiveness of single-cytokine interventions. To date, cytokine-based therapies in periodontitis have largely been investigated in short-term preclinical animal models, with limited translation into well-designed human clinical trials. Given the chronic nature of periodontitis, critical questions remain regarding optimal delivery frequency, route of administration, long-term safety, and therapeutic sustainability. Furthermore, recent evidence over the past few years remains sparse, and reports of limited efficacy and potential challenges underscore the need for more rigorous, long-term clinical investigations (59, 60).

### 3.5 Probiotics

Probiotic microorganisms have emerged as potential adjunctive agents in the management of periodontal disease due to their capacity to modulate both the oral microbiota and host immune response. Preclinical investigations demonstrate that certain probiotic strains can transiently alter the subgingival microbial ecology toward a composition compatible with health, although this effect typically ceases following discontinuation of therapy, likely reflecting the limited persistence of non-oral probiotic strains in the oral cavity (61). Animal studies provide proof-of-concept for host modulation: the gastric administration of *Lactobacillus gasseri* suppressed *Porphyromonas gingivalis*-induced inflammation and bone loss, while the topical use of *Lactobacillus brevis* reduced ligature-induced gingival inflammation and gram-negative bacterial counts, in part through suppression of nitric oxide synthesis (62, 63). The existing evidence from limited human trials suggests that probiotics, particularly *L. reuteri*, may offer modest benefits in improving periodontal health (23, 64). While some studies indicate reductions in gingival inflammation and improved clinical outcomes when combined with standard therapy, the findings are constrained by small sample sizes, varied probiotic strains, dosages, and administration methods (65). A recent meta-analysis emphasizes the need for more rigorous, long-term research to confirm the safety and efficacy of probiotics as adjuncts to periodontal treatment (66).

## 4 Delivery of host modulators (biomaterials, gene therapy, microorganisms)

### 4.1 Host modulator delivering systems: biomaterials

#### 4.1.1 The use of micro-needles in drug delivery

Microneedle (MN) technology has recently been adapted for oral mucosal drug delivery, offering a minimally invasive strategy to overcome epithelial barriers and enable localized administration of vaccines, therapeutics, and diagnostic agents. By creating micropores across the stratified squamous epithelium, MNs enhance drug permeation, residence time, and access to antigen-presenting cells, such as dendritic and Langerhans cells, thereby improving both systemic and mucosal immune responses. To treat oral carcinoma, doxorubicin-loaded PLGA-coated MNs have shown efficacy in targeting cancer cells with reduced systemic exposure in an *in vitro* study (67). In periodontitis, antimicrobial-loaded MNs (e.g., incorporating agents such as chlorhexidine or antibiotics) have been shown to effectively suppress periodontal pathogens and disrupt biofilms, thereby contributing to infection control in periodontal defects (68). In parallel, cytokine- or growth factor–loaded MNs (e.g., IL-4, BMPs, PDGF) have been designed to modulate local immune responses, promote macrophage polarization toward a pro-regenerative phenotype, and stimulate osteogenic and angiogenic pathways, thereby accelerating periodontal regeneration *in vivo (*68). Collectively, these findings highlight the potential of MNs to improve oral mucosal drug delivery, though challenges remain regarding stability in the saliva-rich environment, safety, and translation into well-designed clinical studies (69, 70).

#### 4.1.2 Cell-based encapsulation systems

Cell-based encapsulation has emerged as a promising alternative to conventional cytokine therapies, which often require high systemic doses and are limited by short half-life and toxicities. In this strategy, human cells engineered to secrete cytokines (e.g., IL-2) are encapsulated in alginate microparticles that protect them from immune rejection while allowing cytokine diffusion. Once implanted, these “cytokine factories” generate high local cytokine concentrations with minimal systemic exposure, enabling sustained and controlled immune modulation. Preclinical cancer models demonstrated reduced tumor burden, enhanced CD8+ T cell responses, and durable systemic immunity, accompanied by supportive safety data in nonhuman primates (71). Remaining challenges include long-term biocompatibility, the risk of fibrotic encapsulation, and scalability. Future directions will focus on material optimization, multi-cytokine engineering, and integration with combination immunotherapies (71).

### 4.2 Gene therapy

Gene therapy, already approved by the FDA for certain genetic disorders and cancers, has recently been explored as a host-modulating approach in periodontitis. Among cytokine-based strategies, IL-10 gene delivery is particularly promising due to its potent anti-inflammatory and bone-protective effects. Local IL-10 transfer into periodontal tissues provides sustained expression, suppresses pro-inflammatory mediators (IL-1β, IL-6, TNF-α, RANKL, MMP-8), and reduces alveolar bone loss in experimental models (53). In ovariectomized rats, IL-10 plasmid injection attenuated ligature-induced bone resorption, while murine chamber studies showed that IL-10 gene transfer shifted the cytokine profile from pro-inflammatory to anti-inflammatory dominance in response to *P. gingivalis* (55, 72). These findings highlight IL-10 gene therapy as a potential adjunct for periodontal disease, offering site-specific and prolonged effects compared with recombinant proteins. Nonetheless, translation remains limited by transient expression, vector safety concerns, and the need for optimized delivery and dosing strategies (55, 72).

### 4.3 Engineered bacteria as therapeutics

Engineered probiotics have emerged as promising tools for modulating host responses and delivering therapeutic molecules *in situ*. A commensal species, *L. reuteri*, with immunomodulatory and antimicrobial properties, has been shown to influence host health by shaping the microbiota, enhancing epithelial barrier function, and regulating immune responses, with potential applications in gastrointestinal, metabolic, and inflammatory disorders (71). Beyond naturally occurring strains, genetic engineering strategies have focused on endowing bacteria with therapeutic functions (14, 15). A landmark preclinical study demonstrated that *Lactococcus lactis* engineered to secrete IL-10 significantly reduced inflammation in murine colitis models. This approach was translated into a phase I clinical trial, where transgenic *Lactococcus lactis* expressing IL-10 was orally administered to patients with Crohn’s disease, demonstrating feasibility and safety, though therapeutic efficacy remained modest (18). Collectively, these studies illustrate that engineered bacteria can function as living biotherapeutics to deliver cytokines or modulate host–microbe interactions, offering a targeted, localized, and potentially safer alternative to systemic drug administration (18, 19). To the best of our knowledge, no study has yet applied engineered probiotics to target periodontitis. However, given the central role of dysbiosis and inflammation in its pathogenesis, this strategy could represent a novel and promising approach for managing periodontal disease.

## 5 Discussion

There have been established and emerging host-modulation strategies in the context of systemic and oral diseases. With the focus on periodontitis, this review delineates the mechanisms of action and limitations of several HMTs—including NSAIDs, SPMs, sub-antimicrobial dose doxycycline, cytokine-based treatments, and probiotics—highlighting their measurable yet often limited benefits, which are constrained by factors such as short half-life, systemic effects, treatment rebound, and variability in clinical outcomes (11, 33) (**Table 1**). Recently, novel technologies, including microneedle-based delivery systems, cytokine-secreting cell-encapsulation platforms, IL-10-based gene therapy, and engineered bacterial therapeutics, have been utilized in HMTs (23, 53, 68). These emerging interventions demonstrate promising efficacy in preclinical models of inflammatory and immune-mediated diseases, but they are still in the early stages of translation to periodontal therapy (73). Future research directions may encompass the optimization of localized delivery systems for enhanced stability and sustained release, refinement of anti-inflammatory mediator or cytokine therapies through combinatorial strategies or improved vectors, development of engineered probiotics capable of targeted colonization and *in situ* therapeutic molecule production, integration of microbial and immune-modulatory approaches, execution of long-term clinical trials to assess safety and durability, and the resolution of regulatory and safety challenges related to gene- and microbe-based therapeutics.

**Table 1:** Summary of host-modulation approaches in periodontitis.

Host-modulation strategy	Primary mechanism	Key experimental/clinical findings	Major limitations
Nonsteroidal Anti-Inflammatory Drugs	COX inhibition → ↓ PGE2 → reduced vasodilation and RANKL-mediated bone resorption (36)	Adjunctive use with SRP improves gingival inflammation, CAL, and bone loss in animal and human periodontitis studies (8–10)	Rebound after discontinuation; long-term risks (GI irritation and CVD); it is not suitable for chronic use (11)
Specialized Pro-Resolving Mediators	Promote resolution of inflammation; regulate neutrophil/macrophage/T-cell/B-cell activity; enhance tissue repair (42)	RvE1 significantly inhibits periodontal tissue/bone destruction *in vivo*; promotes PDL and bone regeneration; BLXA4 mouthwash can reduce gingival inflammation (40, 41, 43)	Limited human evidence; lack of long-term data; dosing and delivery not standardized (43)
Sub-Antimicrobial Dose Doxycycline	MMP inhibition (MMP-8, MMP-13) → reduced collagen breakdown (44–46)	FDA-approved it as an adjunct therapy for periodontitis; ~0.3–0.4 mm CAL gain beyond SRP; no significant microbiome or resistance shifts up to 12 months (44–46)	Narrow mechanism; limited long-term data; compliance, cost, and systemic exposure concerns (47)
Cytokine-Targeting Therapies (Anti-IL-1, Anti-TNF-α, Anti-IL-17; IL-10)	Neutralization of pro-inflammatory cytokines or delivery of anti-inflammatory cytokines (50–52)	Inhibiting pro-inflammatory cytokines reduces bone loss in animal periodontitis models; IL-10 increases OPG, reduces RANKL, suppresses inflammatory mediators, and promotes osteoblastic differentiation (53)	Redundant cytokine networks; potential immunosuppression; unclear periodontal clinical applicability (59)
Probiotics	Modulate oral microbiota; regulate host immune responses (61)	Animal models show reduced inflammation and bone loss (*Lactobacillus gasseri*, *Lactobacillus brevis); Limosilactobacillus reuteri* shows modest clinical benefit with SRP (23, 64)	Strain-specific effects; transient colonization; small heterogeneous clinical trials; insufficient long-term data (66)
Microneedle Delivery Systems	Localized mucosal delivery of antimicrobials, cytokines, or growth factors (45)	Antimicrobial microneedles suppress or stimulate pathogens; cytokine/growth-factor microneedles (IL-4, BMPs, PDGF) enhance immune modulation and regeneration in the animal periodontitis model (68)	Stability and safety on oral mucosa; device durability, and absence of clinical trials (69, 70)
Gene Therapy (IL-10 delivery)	Sustained in-tissue expression of anti-inflammatory cytokines (53)	IL-10 reduces inflammatory cytokines, MMPs, and bone loss; shifts to an anti-inflammatory profile in multiple models (55, 72)	Vector safety; transient expression; optimized dosing/delivery required; no clinical translation in periodontitis yet (55, 72)

BLAX4, methyl ester-bezo-lipoxin A4; BMP, Bone morphogenetic protein; CAL, Clinical attachment level; COX, Cyclooxygenase; CVD, Cardiovascular disease; FDA, Food and Drug Administration; GI, Gastrointestinal; IL, Interleukin; MMP, Matrix metalloproteinase; OPG, Osteoprotegerin; PDGF, Platelet-derived growth factor; PDL, Periodontal ligament; PGE2, Prostaglandin E2; RANKL, Receptor activator of nuclear factor-kappa B ligand; RvE1, Resolvin E1; SRP, Scaling and root planing; TNF-α, Tumor necrosis factor-alpha.
